# Meaning in life as a mediator of dark triad with confidence in treatment and subjective evaluation of treatment outcome among male drug abstainers

**DOI:** 10.3389/fpsyt.2022.928101

**Published:** 2022-07-25

**Authors:** Liping Shi, Shijin Sun, Xueli Zhu, Yaoguo Geng

**Affiliations:** ^1^Department of Psychology, School of Social Development and Public Policy, Fudan University, Shanghai, China; ^2^Beijing Key Laboratory of Applied Experimental Psychology, National Demonstration Center for Experimental Psychology Education (Beijing Normal University), Faculty of Psychology, Beijing Normal University, Beijing, China; ^3^School of Education, Zhengzhou University, Zhengzhou, China

**Keywords:** dark triad, substance abuse, confidence in treatment, subjective evaluation of treatment outcomes, meaning in life

## Abstract

**Background:**

Although the roles of personality in predicting substance abuse have been widely documented, few studies have investigated the relationships the dark triad (DT) personalities had with confidence in treatment (CIT) and subjective evaluation of treatment outcome (SETO) in drug abstainers.

**Objective:**

This study examined the relationship between DT and treatment-relevant variables, and the potential effect of meaning in life (MIL) in these links.

**Methods:**

Participants were male inpatients who started substance abuse treatment between June and December 2018 in Henan Province, China. The inclusion criteria were the diagnosis of substance use disorders. The exclusion criteria were illiteracy, comorbidity with psychopathology disorders, intellectual disability, and refusal of consent. A total of 236 men (aged 21–62 years, M = 45.30, SD = 7.72) were randomly selected and reported their DT, MIL, CIT, and SETO.

**Results:**

Results showed that DT was negatively correlated with MIL, CIT, and SETO. MIL was positively correlated with CIT and SETO. The dark triad is associated with CIT both directly and indirectly *via* MIL. DT is indirectly correlated with SETO *via* MIL. Higher levels of DT in drug abstainers can reduce CIT and SETO by decreasing individual's MIL.

**Conclusion:**

This study provides insights into the links between the DT and treatment-relevant variables, which can potentially impact the effectiveness of current substance abuse treatment programs.

## Introduction

Substance abuse has become a serious global problem, affecting approximately 5% of the global adult population (1). Even more worrying, 29.5 million adults worldwide are suffering from drug dependence and need treatment. Substance abuse has ensued significant personal, familial, and economic consequences (1). For example, an estimated 28 million “healthy” life lost worldwide in 2015 as a result of premature death and disability caused by substance abuse. However, the effectiveness of current substance abuse treatment programs is questioned due to the high rate of relapse after treatment (2). Thus, clarifying the risk factors for the recurrence of substance abuse is imperative.

Numerous studies have consistently found that personality traits are associated with the occurrence of substance abuse behaviors, as well as recovery frustration (3, 4). For example, drug users (who use heroin and cocaine) are found to generally show higher neuroticism and lower conscientiousness of Big Five personality traits than healthy controls and smokers (5). Neuroticism may be a risk factor for the recurrence of substance abuse, while conscientiousness is a protective factor for recovery (2).

In comparison with the Big Five personality traits, the dark triad (DT) personality was considered to be part of the dark side of human nature (6). DT consists of three conceptually distinct but empirically overlapping personality traits, namely, Machiavellianism (marked by scheming and manipulative), narcissism (featured by grandiosity, vanity, and entitlement), and psychopathy (characterized by impulsiveness, callousness, and aggression). Dark triad theory has aroused much scientific interest in the past two decades. However, to our knowledge, only a small number of studies addressed the links between DT and substance abuse (5, 7).

For example, DT was found to be positively associated with the frequency of substance use (7), and narcissism and psychopathy were found to be predictors of the number of substances used among college students (5). Some specific aspects of DT, such as disagreeableness, antagonism, impulsivity/deficit in self-control, and sensation-seeking, emerged as predictors for the development of substance use disorders (8–11). Additionally, studies suggest that people high in DT traits have a preference for central nervous system stimulants (e.g., cocaine, caffeine, and methamphetamine), while professional athletes high in DT traits were more likely to use performance-enhancing drugs than those athletes low in DT traits (7).

Although previous studies are influential in underlining the impact of DT on the vulnerability or propensity of substance abuse, little is known about the role of DT in the process of recovery from substance abuse or drug addiction, especially for the impact on treatment-relevant variables, such as confidence in treatment (CIT) and subjective evaluation of treatment outcome (SETO) (12, 13). Confidence in treatment is an important prognostic indicator for the treatment of substance abuse disorder (14), reflecting patients' sense of self-efficacy, an expectation of cure (12), and the degree to refuse drugs. Strong confidence in refusing drugs could significantly reduce the frequency of marijuana use (15) and was a predictor of rehabilitation and long-term treatment effect (15–17). Similar to confidence in treatment, subjective evaluation of treatment outcome is often used to assess the therapeutic effect in the medical field (13) and may be influenced by disease-specific (such as severity) or patient-specific characteristics (such as age, gender, and personality traits). Unfortunately, to date, no work has addressed the links between DT traits and confidence in the treatment and subjective evaluation of treatment outcome.

Previous research has noted associations between substance abuse and meaning in life (the extent to which respondents perceive their lives as meaningful and the extent to which respondents are actively seeking meaning in their lives) (18–22). For example, Frankl reported that people would feel empty if they failed to find meaning in their lives, and then reduced the sense of emptiness through drugs (19). Recently, Zhuang and Chen assessed the effectiveness of the Logotherapy group counseling in alleviating the relapse tendency in a Chinese drug abstainer sample and found that Logotherapy group counseling could effectively reduce the chance of relapse by improving their meaning of life (23). It appears that substance abuse is correlated with the absence of reasons or meaning in life (20), and search and acquisition of meaning in life is essential to successfully abstain from drugs and maintain the long-term curative effect for drug abstainers.

Recently, meaning in life has also been shown to be associated with DT. For example, Wang et al. reported that DT was negatively correlated with meaning in life, and patience fully mediated the links (24). Moreover, Geng et al. found that the sense of life meaning can buffer the influence of early childhood adversity on DT, with the higher the sense of life meaning, the weaker the effects of childhood adversity on life-history strategy and life-history strategy on DT (25). Therefore, we believe a low level of meaning in life might be a psychological marker of those captured by high DT. Based on the above literature, we presume that meaning in life mediates the impact of the dark triad on treatment-relevant variables.

In this study, using Chinese male drug abstainers as subjects, we conducted research to examine the associations between DT and confidence in treatment to fill several gaps in the literature. Based on the nature of DT (such as lower agreeableness, emotional coldness, self-promotion, and a fast life history strategy) and its impact on social-psychological functions (such as lower intimacy in personal relationships, a stronger disposition to aggression and impulsivity, cyberbullying, increased sexual harassment tendency, sleep dysfunctions, as well as disordered eating style; (6, 26–28)), we first hypothesized that DT would be inversely correlated with confidence in treatment. Besides, we also sought to investigate the relationship between DT and subjective evaluation of treatment outcomes in a sample of Chinese male drug abstainers. In view of the fact that there is no relevant literature published, we make no assumptions about the relationship between DT and subjective evaluation of treatment outcome. Moreover, little research has focused on the associations among DT, meaning in life, and treatment-relevant variables so far. Thus, we aimed to examine the associations among DT, meaning in life, confidence in treatment and subjective evaluation of treatment outcome, and expand on previous works by examining the potential mediating effect of meaning in life on the associations between DT and confidence in treatment, as well as subjective evaluation of treatment outcome. It should be noted that, as an exploratory study, the DT total score was first used to evaluate the associations among dark personalities, meaning in life, and treatment relevant variables in this study. This work is expected to provide some guidance for the formulation and implementation of drug treatment policies.

In summary, we expected to find the following: (a) negative correlations between DT and meaning in life, and confidence in treatment; (b) positive correlations between meaning in life and confidence in treatment; and (c) meaning in life serves as a mediator between DT and confidence in treatment.

## Method

### Participants

Participants were male inpatients who started substance abuse treatment between June and December 2018 in Henan Province, China. A total of 236 men with substance use disorders (aged 21–62 years, M = 45.30, SD = 7.72) were diagnosed according to DSM-V criteria. The diagnosis was performed or identified in the department of psychiatry at the 8th people's hospital of Zhengzhou through a non-structured interview and an extensive psychiatric assessment conducted by two qualified psychiatrists. The patients had no illiteracy, intellectual disability, and other major psychiatric co-morbidity. There were 75.74% respondents with education at or above junior high school level, 22.46% were unmarried, 42.37% were married, and 35.17% were divorced, separated, or widowed. All participants entered treatment for the first time and completed our survey during the recovery phase. Most participants received treatment for heroin addiction, but addiction to crystal methamphetamine and cannabis was also represented in the sample. This study was granted in June 2018 by the Ethical Committee of Zhengzhou University and carried out in accordance with the approved guidelines and regulations. All subjects confirmed their compliance through written informed consent.

### Measures

The Short Dark Triad Scale—Chinese version (SD3-C) was adopted to measure the three aspects of the Dark Triad (29). The first nine items identify Machiavellianism (e.g.,“I like to use clever manipulation to get my way”), the second nine items focus on psychopathy (e.g., “People who mess with me always regret it”), and the last nine items measure narcissism (e.g.,“I insist on getting the respect I deserve”). Participants rated the extent to which they agreed with each item on a 5-point scale (1 = strongly disagree, 5 = strongly agree). We calculated each aspect by adding its corresponding items as well as composited a total score of DT by adding all items. In the current study, the scale has sound reliability: α_(Fullscale)_ = 0.85, α_(Machiavellianism)_ = 0.71, α_(psychopathy)_ = 0.71, and α_(narcissism)_ = 0.63. Only the DT total score was used to evaluate the associations among dark personalities, meaning in life, and treatment-relevant variables in this study.

The 10-item Meaning in Life Questionnaire (MLQ) is a self-reported inventory designed to measure perceived life meaning (30). In the current study, we utilized the Chinese version of MLQ (31), which comprises two subscales: presence and search. Items anchored on a 7-point scale range from one (absolutely untrue) to seven (absolutely true). Presence assesses the degree to which life is perceived as meaningful (e.g., “My life has a clear sense of purpose”), while Search assesses individual's motivation to discover meaning in life (e.g., “I am always looking to find my life's purpose”). We calculated each subscale by adding its corresponding items. In this study, the internal consistency reliability of the entire scale was 0.75 for the entire questionnaire, 0.69 for Presence subscale, and 0.77 for Search subscale.

The Confidence in Treatment (CIT) was assessed with five items derived from the Treatment Motivation Questionnaire (TMQ) (32). Items were rated from 1 (not true at all) to 4 (very true). Items were added to create an indicator of CIT. A higher score represents a higher sense of confidence in treatment. In our sample, the internal consistency reliability of the entire scale was 0.66.

Subjective evaluation of treatment outcome (SETO) was measured with one item (i.e., “I believe that the treatment provided is effective”) on a 5-point scale (1 = strongly disagree, 5 = strongly agree). When the item clears the conceptual ambiguity and reflects the concept common to participants' daily experiences, single-item measures can be appropriated (33). We had explained the meaning of this item description, so we hope we can obtain an accurate indicator of SETO. A higher value stands for a higher evaluation of SETO.

### Statistical analysis

To check our hypotheses, correlation analysis was performed by SPSS 23.0 and our proposed structural equation model (SEM) was examined by the AMOS 22.0 program with DT and meaning in life as latent variables and confidence in treatment and subjective evaluation of treatment outcome as observed variables. DT served as the predictor variables, confidence in treatment and subjective evaluation of treatment outcome as the outcome variables, and meaning in life as the mediator. To determine whether an indirect effect or a total effect was statistically significant, we used maximum likelihood estimation and the bias-corrected bootstrap 95% confidence interval (*CI*) based on 5,000 bootstrapping. Several indices were considered to evaluate the adjustment of the model (i.e., the minimum discrepancy (χ^2^/*df* ; values in the range of 2–5 are indicative of an acceptable fit) (34), the Comparative Fit Index and the Incremental Fit Index (CFI and IFI; values equal to or over 0.95 indicate a good adjustment), and root mean square error of approximation (RMSEA; values below 0.08 indicate a good fit) (35).

## Results

### Descriptive analyses

In Table 1, we reported the descriptive statistics and correlations among study variables. The composite of DT was negatively correlated with confidence in treatment, subjective evaluation of treatment outcomes, and meaning in life. In addition, meaning in life, confidence in treatment, and subjective evaluation of treatment outcomes were positively correlated with each other.

**Table 1:** Descriptive statistics and correlations of study variables.

	***Mean***	***SD***	**1**	**2**	**3**	**4**	**5**	**6**	**7**	**8**	**9**
1 Dark triad	72.65	13.27	1								
2 Machiavellianism	25.71	6.17	0.80^**^	1							
3 Narcissism	24.29	4.65	0.69^**^	0.30^**^	1						
4 Psychopathy	22.62	6.06	0.85^**^	0.52^**^	0.44^**^	1					
5 MIL	48.65	11.72	−0.16^*^	−0.10	0.05	−0.28^**^	1				
6 Presence	21.99	6.22	−0.14^*^	−0.10	0.04	−0.22^**^	0.89^**^	1			
7 Search	26.66	6.83	−0.15^*^	−0.08	0.06	−0.28^**^	0.91^**^	0.62^**^	1		
8 CIT	12.83	4.04	−0.14^*^	−0.13	−0.04	−0.16^*^	0.16^*^	0.14^*^	0.15^*^	1	
9 SETO	3.89	1.19	−0.16^*^	−0.10	−0.05	−0.22^**^	0.27^**^	0.27^**^	0.20^**^	0.24^**^	1

* p < 0.05 and

** p < 0.01.

MIL, Meaning in life; CIT, confidence in treatment; SETO, subjective evaluation of treatment outcomes.

### Structural equation modeling

**Figure 1:**
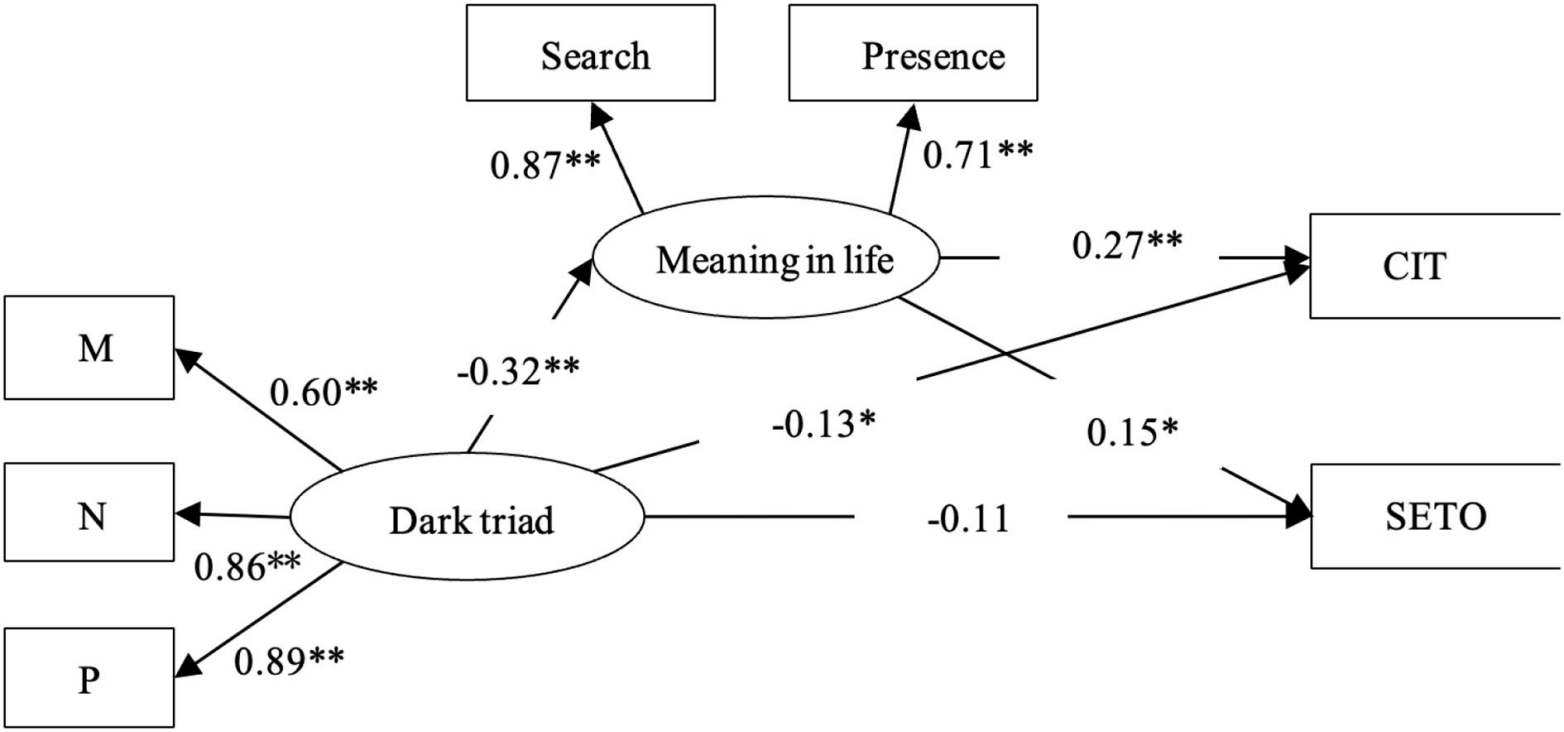
The mediated model of meaning in life between DT and CIT and SETO. CIT, confidence in treatment; SETO, subjective evaluation of treatment outcomes. Numbers are standardized regression coefficients. ^*^p < 0.05, ^**^p < 0.01.

The results of SEM indicated an acceptable fit to the data (χ^2^/*df* = 2.08, CFI = 0.96, IFI = 0.96, and RMSEA = 0.07). Analysis of parameter estimates indicated that except for the pathway from DT to subjective evaluation of treatment outcomes, standard coefficients of pathway were all significant (as shown in Figure 1), indicating that DT predicts subjective evaluation of treatment outcomes indirectly *via* meaning in life (β = −0.05, SE = 0.03, 95% *CI* [−0.11, −0.01]), and *R*^2^ = 0.24; while DT can predict confidence in treatment both directly (β = −0.13, SE = 0.07, 95% *CI* [−0.27, −0.03]) and indirectly (β = −0.09, SE = 0.03, 95% *CI* [−0.15, −0.04]), and *R*^2^ = 0.74.

## Discussion

For all we know, this is the first study that mainly focused on the relationships among the DT, treatment-relevant variables, and meaning in life. The results revealed that DT was negatively correlated with confidence in treatment and subjective evaluation of treatment outcomes. Meaning in life was positively correlated with confidence in treatment and subjective evaluation of treatment outcomes. DT can predict confidence in treatment both directly and indirectly through meaning in life, while DT predicts subjective evaluation of treatment outcomes indirectly.

In support of our hypotheses, DT was found to be negatively correlated with confidence in treatment and subjective evaluation of treatment outcomes, suggesting that drug abstainers scoring high on DT are more likely to have low confidence in treatment and low subjective evaluation of treatment outcomes. There are theoretical reasons and empirical evidence for these findings. Theoretically, DT may be an indicator of a fast Life History strategy (8, 36, 37). According to the Life History Theory (38), individuals need to trade-off reproductive efforts against somatic efforts continually, and these trade-offs will stabilize into behavioral strategies that vary in a continuum from slow to fast. A fast life strategy is reflective of reproductive efforts outweighing somatic efforts, and is mainly manifested by casual sex, short-term mating, risk-taking behaviors (such as substance use), and a lack of long-term planning and responsibility (39, 40). Individuals who (e.g., those with a high level of DT) follow a fast strategy go at the expense of psychological and physical health to solve adaptive tasks.

On the other hand, empirical studies have revealed that DT is marked by failure to self-regulate, risky behavior, dissatisfaction of the need, and reduced subjective wellbeing (41–43). Therefore, drug abstainers with a high level of DT traits tend to pay less attention to improving healing and commit to treatment, and they consequently perceive less fulfillment and satisfaction, thus following worse treatment evaluation.

As expected, the present study was able to provide an insight into the underlying psychological process that may be associated with treatment-relevant variables. SEM revealed that DT indirectly lowered confidence in treatment and subjective evaluation of treatment outcomes through meaning in life. These results suggest that (1) the greater their scores on DT, the more participants endorsed a lower level of meaning in life; (2) the lower their scores on meaning in life, the less participants endorsed confidence in treatment and subjective evaluation of treatment outcome; and (3) meaning in life mediated the effects of DT on confidence in treatment and subjective evaluation of treatment outcome. In this sense, meaning in life may be an important basic psychological function to understand the effects of personality on relapse tendency (44). These results are largely in line with previous studies on the relationships between DT and internalizing problems (such as less appreciation of others and increased negative evaluation) (26).

There are several theoretical and practical contributions and implications of this study. First, we are interested in the relationship between DT and substance abuse treatment. For one thing, our findings enrich the literature about the relevant variables of substance abuse treatment in Chinese culture. Related studies have concentrated on the Big Five personality traits and largely ignored the impact of DT (2). Combined with previous studies, our findings provide a more complete picture by including the DT as a factor that may relate to confidence in substance abuse treatment and subjective evaluation of treatment outcome. The results provide initial evidence of negative relations between the DT and substance abuse treatment variables. Another reason is that the negative effects of the DT on substance abuse treatment further indicate that the DT has a broader scope of impacts because the dark side of the DT has mainly been limited to the susceptibility and severity of drug misuse in previous studies (7).

Second, this study explored the possible indirect pathways involving emotional function and observed that the DT weakened meaning in life, which could then damage confidence in treatment and subjective evaluation of treatment outcome. Substance abuse treatment is not only a personal disturbance but also a social issue. These findings could offer some guidance for the government of China and other courtiers to set and implement policies for improving drug treatment. For example, in the process of detoxification, relevant staff should pay attention to the plasticity of personality and the impact of a dark personality on life meaning. Focusing on reducing the negative and destructive characteristics of drug abstainers, and helping them to gain and strengthen meaning should be treated as ways to improve recovery and reduce relapse.

## Limitation and future directions

Despite the strengths of the current study, its potential limitations should be considered. First, the cross-sectional nature of our data limits our ability to draw conclusions about causal links. Future studies should therefore make use of longitudinal study designs. Second, we must pay attention to the problem that the effect sizes are too small in predicting CIT and SETO, which means that there are other key mediating variables, and future research should explore the other internal mechanisms. Third, as a Chinese drug abstainer sample was studied, the treatment pattern of drug abstainers was different from other mental disorders. Future research would generalize our findings to a diverse clinical population. Fourth, given that Machiavellianism, narcissism, and psychopathy include their own dimensions, SD3 may be too brief to capture the whole picture of the DT. Future research may examine the associations between meaning in life, confidence in treatment, and subjective evaluation of treatment outcomes, and different types of psychopathy, narcissism. Lastly, we draw conclusions only based on the Chinese population. Future research should consider a more varied cultural populations, such as Western populations.

## Conclusion

Taken together, the present study underscores the importance of the DT personality as a destructive factor of confidence in treatment and subjective evaluation of treatment outcomes, and meaning in life as a protective factor of confidence in treatment and subjective evaluation of treatment outcomes.

## Data availability statement

The raw data supporting the conclusions of this article will be made available by the authors, without undue reservation.

## Ethics statement

The studies involving human participants were reviewed and approved by Ethical Committee of Zhengzhou University. The patients/participants provided their written informed consent to participate in this study.

## Author contributions

LS and YG conceptualized and designed the study. SS gave directions to the study. LS and XZ analyzed and interpreted the data and wrote the manuscript. SS, XZ, and YG critically reviewed the manuscript text. All authors reviewed the manuscript and given final approval of the version to be published.

## Funding

This work was supported by the Education Program of the National Social Science Fund of China (BBA170064).

## Conflict of interest

The authors declare that the research was conducted in the absence of any commercial or financial relationships that could be construed as a potential conflict of interest.

## Publisher's note

All claims expressed in this article are solely those of the authors and do not necessarily represent those of their affiliated organizations, or those of the publisher, the editors and the reviewers. Any product that may be evaluated in this article, or claim that may be made by its manufacturer, is not guaranteed or endorsed by the publisher.
